# Draft Genome Sequence of *Halomonas* sp. Strain ML-15, a Haloalkaliphilic, Polycyclic Aromatic Hydrocarbon-Degrading Bacterium

**DOI:** 10.1128/MRA.01175-20

**Published:** 2020-11-19

**Authors:** Mitchell Henry Wright, Steven Robert Bentley, Anthony Carlson Greene

**Affiliations:** a School of Environment and Science, Nathan Campus, Griffith University, Brisbane, Queensland, Australia; b Leviathan Biosciences, Brisbane, Queensland, Australia; c Griffith University DNA Sequencing Facility, Brisbane, Queensland, Australia; Georgia Institute of Technology

## Abstract

*Halomonas* sp. strain ML-15 is an aerobic, haloalkaliphilic bacterium capable of degrading polycyclic aromatic hydrocarbons (PAHs). The draft genome sequence of the isolate contains 19 contigs encompassing 4.8 Mb and a G+C content of 65.38%. This sequence will provide essential information for future studies of PAH degradation, particularly under haloalkaliphilic conditions.

## ANNOUNCEMENT

Wastes generated from incomplete combustion of carbon-containing material and fossil fuels often contain toxic and hazardous organic compounds such as polycyclic aromatic hydrocarbons (PAHs) (1). Under aerobic conditions and with essential nutrients, microorganisms can transform many PAHs into less toxic compounds, often mineralizing them to carbon dioxide, water, and cell biomass (2). While these processes are well studied at circumneutral pH and in freshwater systems, very little work to date has focused on haloalkaliphilic PAH degradation. Several *Halomonas* strains have been found to degrade PAHs (3, 4), though none have been haloalkaliphilic. The aim of the study associated with *Halomonas* sp. strain ML-15 was to assess PAH degradation by haloalkaliphilic bacteria, focusing on both the rates of degradation and the genes associated with these processes.

*Halomonas* sp. strain ML-15 was first isolated in April 2012 from water samples obtained from Mono Lake, California, in late November 2008 (stored at 4°C until used). The bacterium was initially grown in artificial Mono Lake (AML) medium, formulated to replicate haloalkaliphilic conditions in Mono Lake as previously described (5) and amended with 2 mM anthracene and 0.1 g/liter yeast extract as sources of carbon. Strain ML-15 was isolated using serial streaking from an enrichment culture positive for growth (5). Initial identity was determined through 16S rRNA gene sequencing using methods previously described (6) and placed the isolate closest to Halomonas socia (97.8% similarity).

For genomic sequencing, strain ML-15 was cultivated overnight in liquid medium (6). Purified DNA was extracted as previously described (7) and subsequently prepared for sequencing. Briefly, 450 ng of the DNA underwent fragmentation by sonication, followed by isolation of 800-bp fragments by electrophoresis. The ends of these products were repaired and prepared for ligation of the Illumina flow cell adapters and unique indexes. These products were sequenced using paired-end 150-bp reads on an Illumina HiSeq 4000 sequencer (Omics2view, Germany). This resulted in 8,480,448 reads, equating to 1.27 Gbp of data. The following bioinformatic analysis used default parameters unless otherwise stated. Trimmomatic v0.39 (8) was used to retain reads of >100 bp after trimming nucleotides with a quality of <Q30 from the start, end, and in a 4-bp sliding window. From these reads, those that aligned to the Illumina PhiX genome sequence or the UniVec v10.0 database of contaminating vector sequences using Bowtie 2 v2.3.5.1 were omitted. The resultant 6,189,544 reads then underwent *de novo* assembly using Velvet v1.2.10 (9), with the expected insert size set to 500 bp. A series of k-mer lengths were assessed, including lengths of 31, 51, 71, 91, 111, and 131. Contigs with <1,000 nucleotides and a read-pair count of less than 10 were omitted. Assemblies were compared using QUAST v5.0.2 (10). Taxonomic identification was conducted and annotations performed using the Prokaryotic Genome Annotation Pipeline (PGAP) v4.13 (11).

The 111-kmer *de novo* assembly of strain ML-15 produced 19 contigs encompassing 4,802,587 bp, with a G+C content of 63.38%. The largest contig was 2,269,676 bp, and the *N*_50_ and *L*_50_ values were 436,491 bp and 2, respectively. This assembly was identified to be 99.7% complete from 619 single-copy orthologs in the *Oceanospirillales* lineage using BUSCO v4.1.4. Annotation suggested that the assembly contained 4,496 genes, 5 complete rRNAs (3 5S, 1 16S, 1 23S), and 62 tRNAs. Further, only 43 pseudogenes were identified; 19 of these were due to incomplete coverage. Additionally, one CRISPR array was identified. Average nucleotide identity analysis from PGAP suggests that isolate ML-15 does not align closer than 90% with other bacterial genomes, with the highest percent identity to Halomonas pantelleriensis of 87.5%.

### Data availability.

The genome sequence and annotation data for *Halomonas* sp. strain ML-15 were deposited in DDBJ/GenBank under BioProject number PRJNA664264, BioSample number SAMN16203731, SRA number SRS7417957, and the accession number JACXZT000000000. The version described in this paper is version JACXZT010000000.
